# Screening of preoperative obstructive sleep apnea by cardiopulmonary coupling and its risk factors in patients with plans to receive surgery under general anesthesia: a cross-sectional study

**DOI:** 10.3389/fneur.2024.1370609

**Published:** 2024-07-24

**Authors:** Shujie Hou, Guojia Zhu, Xu Liu, Chuan Wang, Junchao Liang, Wei Hao, Lili Kong

**Affiliations:** ^1^Graduate School of Hebei University of Traditional Chinese Medicine, Shijiazhuang, China; ^2^School of Basic Medicine, Hebei University of Traditional Chinese Medicine, Shijiazhuang, China; ^3^Department of Anesthesiology and Perioperative Medicine, Hebei Provincial Hospital of Traditional Chinese Medicine, Shijiazhuang, China

**Keywords:** preoperative obstructive sleep apnea, cardiopulmonary coupling, heart rate variability, prevalence, risk factor

## Abstract

**Objective:**

Preoperative obstructive sleep apnea (OSA) is supposed to be the abnormally high occurrence of OSA the night before surgery under general anesthesia. This study aimed to evaluate the prevalence preoperative OSA using cardiopulmonary coupling (CPC) and its correlation with imbalance of sympathetic/parasympathetic nervous system.

**Methods:**

A total of 550 patients with plans to receive surgery under general anesthesia were enrolled. All patients were assigned to wear CPC on the night before surgery until the next day. Sleep quality characteristics, heart rate variation parameters, and apnea-hypopnea index were acquired. The diagnosis of pre-existing OSA was not considered in the current study.

**Results:**

According to apnea-hypopnea index, 28.4%, 32.2%, 26.2%, and 13.3% patients were assessed as no, mild, moderate, and severe operative OSA, respectively. Multivariate logistic regression model revealed that higher age [*p* < 0.001, odds ratio (OR) = 1.043] was independently and positively associated with preoperative OSA; heart rate variation parameters representing the imbalance of sympathetic/parasympathetic nervous system, such as higher low-frequency (*p* < 0.001, OR = 1.004), higher low-frequency/high-frequency ratio (*p* = 0.028, OR = 1.738), lower NN20 count divided by the total number of all NN intervals (pNN20; *p* < 0.001, OR = 0.950), and lower high-frequency (*p* < 0.001, OR = 0.998), showed independent relationships with a higher probability of preoperative OSA. Higher age (*p* = 0.005, OR = 1.024), higher very-low-frequency (*p* < 0.001, OR = 1.001), and higher low-frequency/high-frequency ratio (*p* = 0.003, OR = 1.655) were associated with a higher probability of moderate-to-severe preoperative OSA, but higher pNN10 (*p* < 0.001, OR = 0.951) was associated with a lower probability of moderate-to-severe preoperative OSA.

**Conclusion:**

Preoperative OSA is prevalent. Higher age and imbalance of sympathetic/parasympathetic nervous system are independently and positively associated with a higher occurrence of preoperative OSA. CPC screening may promote the management of preoperative OSA.

## 1 Introduction

Obstructive sleep apnea (OSA) is a prevalent disorder of the respiratory system caused by the recurrent narrowing or collapse of the airway during sleep (1, 2). OSA is associated with male sex, higher age, overweight or obesity, and consumption of cigarettes and alcohol (3, 4). OSA is considered to induce several possible consequences, including excessive sleepiness, fatigue, metabolic disorders, and cardiovascular diseases (5–7). More importantly, subjects with OSA about to receive surgery under general anesthesia should be properly managed since OSA is associated with adverse postoperative outcomes, such as pulmonary complications, cardiovascular complications, delirium, and even mortality (8–10). Nevertheless, it is generally considered that OSA is largely underdiagnosed (11, 12). Therefore, it is critical to recognize OSA to better manage subjects about to receive surgery under general anesthesia.

Preoperative OSA is supposed to be the abnormally high occurrence of OSA the night before surgery under general anesthesia. Patients about to receive surgery under general anesthesia may have some negative emotions, such as fear of surgery and worry about the surgical outcome (13). These negative emotions could lead to the imbalance of sympathetic and parasympathetic nervous systems; while the imbalance of sympathetic and parasympathetic nervous system is recognized as a critical factor associated with OSA (14, 15). However, the information on preoperative OSA assessment is still lacking.

Cardiopulmonary coupling (CPC) is a technique generating data on heart rate variability and respiration (16). Compared with laboratory-based polysomnography (the gold standard diagnostic modality of OSA), CPC is characterized by convenience and portability (17). Several studies have suggested that CPC shows high performance for the diagnosis of OSA (18, 19). For instance, a study includes subjects undergoing full-night in-laboratory polysomnography and CPC simultaneously; the results reveal software-generated apnea-hypopnea index (AHI) by CPC and AHI data by polysomnography are highly correlated with each other; this study also revealed that the software-generated AHI by CPC show excellent performance for diagnosing OSA, especially severe OSA with sensitivity of 100% and specificity of 93.63% (18). Another study discloses that in children with severe OSA, the AHI from CPC is not different from AHI from polysomnography, suggesting the good accuracy of CPC for diagnosing severe OSA in children (19). Xie et al. (20) report that sensitivity of CPC for recognizing AHI ≥ 5/h, ≥10/h, ≥15/h, ≥20/h, and ≥30/h by polysomnography are 0.82, 0.93, 0.96, and 0.77, respectively, and the specificity are 0.50, 0.75 0.72, 0.80, and 0.86, respectively. Ma et al. (21) show that for AHI ≥ 5/h, ≥ 15/h, and ≥ 30/h by polysomnography, CPC has a sensitivity of 93.8, 92.7 and 89.5%, and specificity of 67.8, 72.2 and 79.8%, respectively.

Therefore, the current study aimed to evaluate preoperative OSA by CPC and its correlative factors in subjects who had plans to receive general anesthetic for elective surgery.

## 2 Methods

### 2.1 Patients

A total of 550 patients who had plans to receive general anesthetic for elective surgery from December 2021 to November 2022 in Hebei Provincial Hospital of Traditional Chinese Medicine were consecutively enrolled in this cross-sectional study. The inclusion criteria contained: (i) had plans to receive general anesthetic for elective surgery; (ii) had the willingness to wear cardiopulmonary coupling (CPC) for sleep quality and heart rate variability (HRV) evaluation; (iii) aged ≥18 years; (iv) Pittsburgh sleep quality index (PSQI) score ≤ 5. The exclusion criteria contained: (i) with a pacemaker; (ii) with cardiac arrhythmias; (iii) with CPC assessment system acquisition validity <80% or with acquisition interruption; (iv) during pregnancy; (v) with severe or acute somatic illnesses in the 3 months prior to enrollment; (vi) addicted to beta-blockers, alcohol, or other psychotropic drugs. In order to reflect the actual status of preoperative OSA, the pre-existing diagnosis of OSA was not considered in the participants. This study was approved by the Ethics Committee of Hebei Provincial Hospital of Traditional Chinese Medicine (No. HBZY2020-KY-067-02). Each patient was informed about the study and signed an informed consent.

### 2.2 CPC assessment system

All patients were forbidden to consume alcohol or caffeine, take a nap, or engage in prolonged or strenuous exercise on the day of monitoring, and a person was assigned to wear a CPC electrocardiogram (ECG) signal recorder (AECG-600D, Nanjing Fengsheng Yongkang Software Technology Co, China, Supplementary Figure 1A). All patients started to record from 21:00–22:00 on the night before surgery, and the instrument was removed at 6:00–7:00 on the next day. The instrument was worn between the left edge of the sternum and the left midclavicular line between the 3rd and 4th intercostal space (above the nipple line against the medial flat); females could wear it between the 2nd and 3rd intercostal spaces due to their physiological composition, and it was attached to the surface of the skin through the adhesive backing (Supplementary Figure 1B). The ECG signal was obtained by the ECG collector, and the result was uploaded to the computer. The system automatically analyzed CPC data and generated the sleep quality report.

### 2.3 Data documentation

Patients’ data were obtained, which included: (i) clinical characteristics: age, gender, body mass index (BMI), education level, hypertension, diabetes, perioperative anxiety scale-7 (PAS-7) (22), and surgical site; (ii) sleep quality characteristics (Supplementary Figure 2A): bedtime, sleep time, sleep efficiency, sleep latency, and acute insomnia; (iii) HRV time domain characteristics (Supplementary Figure 2B): standard deviation (SD) of all normal RR intervals (SDNN), SD of 5 min average normal RR intervals (SDANN), the root mean square of the successive differences (rMSSD), HRV trigonometric index (HRVTI), NN50 count divided by the total number of all NN intervals (pNN50); (iv) HRV frequency domain characteristics (Supplementary Figure 2C): ultra-low-frequency (ULF), very-low-frequency (VLF), low-frequency (LF), high-frequency (HF), and LF/HF ratio.

### 2.4 Preoperative obstructive sleep apnea

Apnea-hypopnea index (AHI) was obtained, based on which the preoperative OSA was assessed: no preoperative OSA, AHI < 5; mild preoperative OSA, 15 > AHI ≥ 5; moderate preoperative OSA, 30 > AHI ≥ 15; and severe preoperative OSA, AHI ≥ 30.

### 2.5 Statistics

SPSS 26.0 (SPSS Inc., United States) was used for statistical analysis. Comparison analyses were conducted using one-way analysis of variance (ANOVA), student t, and Chi-square tests. Correlation analyses were conducted using Pearson’s correlation and Spearman’s rank correlation tests. Independent factors were screened using forward stepwise multivariate logistic and linear regression models. *p*-values < 0.05 were considered significant.

## 3 Results

### 3.1 Baseline characteristics

The enrolled 550 patients had a mean age of 52.3 ± 15.6 years and consisted of 276 (50.2%) males. There were 143 (26.0%) patients with hypertension and 60 (10.9%) patients with diabetes. The mean PAS-7 score was 10.0 ± 4.4 and there were 382 (69.5%) patients assessed as anxiety by PAS-7 score. There were 140 (25.5%) patients with surgery on the head, 83 (15.1%) patients with surgery on the chest, 313 (56.9%) patients with surgery on the abdomen, and 14 (2.5%) patients with surgery on the extremities. The other baseline characteristics were shown in Table 1.

**Table 1:** Clinical characteristics.

Items	Patients (*N* = 550)
Age (years), mean ± SD	52.3 ± 15.6
<60 years, No. (%)	358 (65.1)
≥60 years, No. (%)	192 (34.9)
Male, No. (%)	276 (50.2)
BMI (kg/m^2^), mean ± SD	24.9 ± 3.8
<27 kg/m^2^, No. (%)	382 (69.5)
≥27 kg/m^2^, No. (%)	168 (30.5)
<30 kg/m^2^, No. (%)	494 (89.8)
≥30 kg/m^2^, No. (%)	56 (10.2)
**Education level, No. (%)**	
Low (below high school)	280 (50.9)
High (high school and above)	270 (49.1)
Hypertension, No. (%)	143 (26.0)
Diabetes, No. (%)	60 (10.9)
PAS-7 score, mean ± SD	10.0 ± 4.4
Anxiety by PAS-7 score, No. (%)	382 (69.5)
**Surgical site, No. (%)**	
Head	140 (25.5)
Chest	83 (15.1)
Abdomen	313 (56.9)
Extremities	14 (2.5)

SD, standard deviation; BMI, body mass index; PAS-7, perioperative anxiety scale-7.

### 3.2 Assessment of sleep quality and HRV characteristics

Regarding sleep quality, the mean total, deep, light, and REM sleep times were 7.0 ± 1.6 h, 2.4 ± 1.4 h, 3.0 ± 1.3 h, and 1.7 ± 0.7 h, respectively. The mean sleep efficiency was 75.5 ± 11.3%. The mean AHI score was 15.5 ± 15.5. More detailed characteristics of sleep quality and HRV were shown in Table 2.

**Table 2:** Sleep quality and HRV characteristics.

Items	Patients (*N* = 550)
**Sleep quality characteristics**	
Bedtime (h), mean ± SD	9.4 ± 1.7
Total sleep time (h), mean ± SD	7.0 ± 1.6
Deep sleep time (h), mean ± SD	2.4 ± 1.4
Light sleep time (h), mean ± SD	3.0 ± 1.3
REM sleep time (h), mean ± SD	1.7 ± 0.7
Awakening time (h), mean ± SD	1.7 ± 0.9
First falling asleep time (h), mean ± SD	2.0 ± 7.1
Sleep efficiency (%), mean ± SD	75.5 ± 11.3
Sleep latency (min), mean ± SD	12.1 ± 21.2
Prolongation of sleep latency, No. (%)	73 (13.3)
Acute insomnia, No. (%)	439 (79.8)
AHI, mean ± SD	15.5 ± 15.5
**HRV characteristics**	
SDNN (ms), mean ± SD	112.4 ± 34.1
SDANN (ms), mean ± SD	94.4 ± 31.4
rMSSD (ms), mean ± SD	30.9 ± 17.6
pNN10 (%), mean ± SD	56.6 ± 16.5
pNN20 (%), mean ± SD	37.7 ± 18.4
pNN30 (%), mean ± SD	26.1 ± 19.3
pNN40 (%), mean ± SD	13.5 ± 13.7
pNN50 (%), mean ± SD	9.9 ± 11.9
ULF (ms^2^), mean ± SD	13450.3 ± 9448.0
VLF (ms^2^), mean ± SD	2010.0 ± 1304.2
LF (ms^2^), mean ± SD	753.2 ± 617.2
HF (ms^2^), mean ± SD	667.1 ± 911.6
LF/HF ratio, mean ± SD	1.6 ± 0.8

HRV, heart rate variability; SD, standard deviation; REM, rapid-eye-movement; AHI, apnea-hypopnea index; SDNN, SD of all normal RR intervals; SDANN, SD of 5 min average normal RR intervals; rMSSD, the root mean square of the successive differences; pNN10, NN10 count divided by the total number of all NN intervals; pNN20, NN20 count divided by the total number of all NN intervals; pNN30, NN30 count divided by the total number of all NN intervals; pNN40, NN40 count divided by the total number of all NN intervals; pNN50, NN50 count divided by the total number of all NN intervals; ULF, ultra-low-frequency; VLF, very-low-frequency; LF, low-frequency; HF, high-frequency.

### 3.3 Prevalence of preoperative OSA

**Figure 1:**
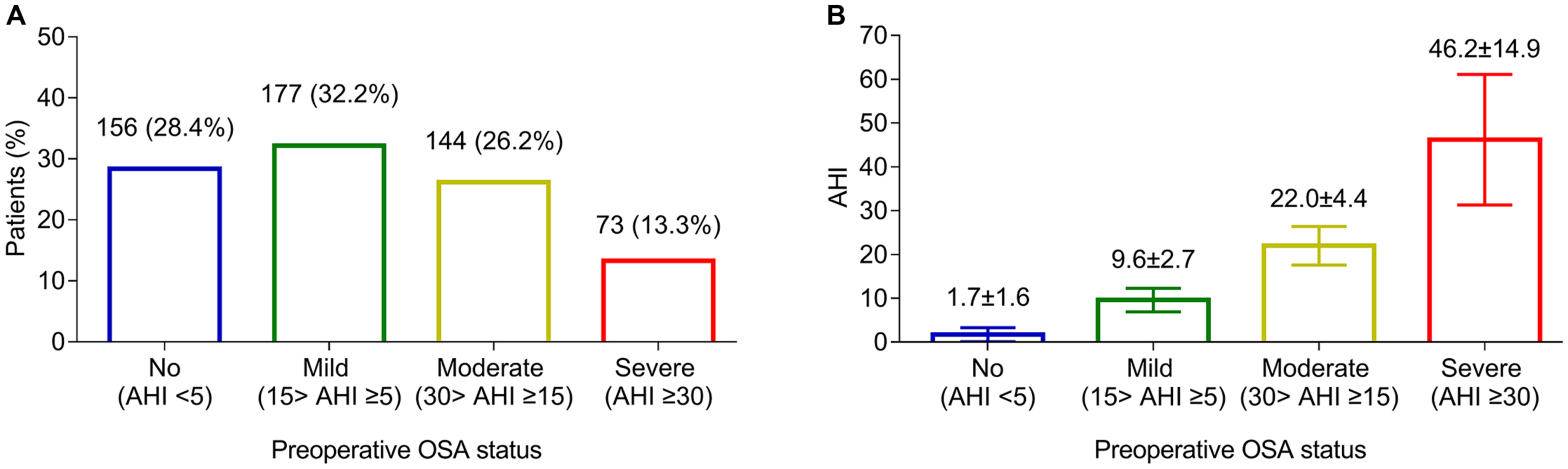
Preoperative OSA assessment. Number and proportion of patients with no, mild, moderate, and severe preoperative OSA **(A)**. The mean AHI in patients with no, mild, moderate, and severe preoperative OSA **(B)**.

There were 156 (28.4%) patients assessed as no preoperative OSA, and the other 394 (71.6%) patients had preoperative OSA. In detail, 177 (32.2%) patients were assessed as mild preoperative OSA, 144 (26.2%) patients were assessed as moderate preoperative OSA, and 73 (13.3%) patients were assessed as severe preoperative OSA (Figure 1A). The mean AHI was 1.7 ± 1.6 in patients without preoperative OSA, 9.6 ± 2.7 in patients with mild preoperative OSA, 22.0 ± 4.4 in patients with moderate preoperative OSA, and 46.2 ± 14.9 in patients with severe preoperative OSA (Figure 1B).

### 3.4 Association of clinical characteristics, sleep quality, and HRV with preoperative OSA

The association of clinical characteristics, sleep quality, and HRV with preoperative OSA, moderate-to-severe preoperative OSA, and severe preoperative OSA is presented in Table 3.

**Table 3:** Correlation of preoperative OSA status with characteristics.

Items	Without preoperative OSA (AHI < 5)	With preoperative OSA (AHI ≥ 5)			*P1* value	*P2* value	*P3* value	*P4* value	*P5* value
		Mild preoperative OSA (15 > AHI ≥ 5)	Moderate preoperative OSA (30 > AHI ≥ 15)	Severe preoperative OSA (AHI ≥ 30)					
**Clinical characteristics**									
Age (years), mean ± SD	48.4 ± 16.4	51.3 ± 14.2	54.0 ± 16.1	59.3 ± 13.4	**<0.001**	**<0.001**	**0.001**	**0.003**	**0.001**
Gender, No. (%)					**<0.001**	**<0.001**	**0.002**	**<0.001**	**0.035**
Female	101 (36.9)	95 (34.7)	54 (19.7)	24 (8.8)					
Male	55 (19.9)	82 (29.7)	90 (32.6)	49 (17.8)					
BMI (kg/m^2^), mean ± SD	24.7 ± 4.0	25.1 ± 3.5	25.0 ± 4.1	25.2 ± 3.4	0.729	0.290	0.909	0.989	0.689
Education level, No. (%)					0.175	0.225	0.175	0.068	0.669
Low	73 (26.1)	84 (30.0)	83 (29.6)	40 (14.3)					
High	83 (30.7)	93 (34.4)	61 (22.6)	33 (12.2)					
Hypertension, No. (%)					0.093	0.065	0.241	0.122	0.201
No	124 (30.5)	134 (32.9)	101 (24.8)	48 (11.8)					
Yes	32 (22.4)	43 (30.1)	43 (30.1)	25 (17.5)					
Diabetes, No. (%)					0.076	**0.015**	0.660	0.380	0.549
No	147 (30.0)	157 (32.0)	124 (25.3)	62 (12.7)					
Yes	9 (15.0)	20 (33.3)	20 (33.3)	11 (18.3)					
PAS-7 score, mean ± SD	10.3 ± 4.4	9.6 ± 4.5	10.0 ± 4.4	10.2 ± 4.4	0.468	0.240	0.565	0.297	0.509
Surgical site, No. (%)					0.579	0.736	0.394	0.840	0.258
Head or chest	65 (29.1)	70 (31.4)	63 (28.3)	25 (11.2)					
Abdomen or extremities	91 (27.8)	107 (32.7)	81 (24.8)	48 (14.7)					
**Sleep quality characteristics**									
Bedtime (h), mean ± SD	9.1 ± 1.6	9.4 ± 1.7	9.5 ± 1.7	9.5 ± 1.8	0.232	**0.040**	0.963	0.801	0.833
Total sleep time (h), mean ± SD	7.2 ± 1.5	7.1 ± 1.6	7.0 ± 1.5	6.8 ± 1.8	0.397	0.293	0.407	0.219	0.310
Deep sleep time (h), mean ± SD	3.6 ± 1.3	2.6 ± 1.1	1.7 ± 0.9	0.8 ± 0.8	**<0.001**	**<0.001**	**<0.001**	**<0.001**	**<0.001**
Light sleep time (h), mean ± SD	2.0 ± 0.8	2.8 ± 0.8	3.5 ± 1.0	4.7 ± 1.4	**<0.001**	**<0.001**	**<0.001**	**<0.001**	**<0.001**
REM sleep time (h), mean ± SD	1.6 ± 0.7	1.8 ± 0.7	1.7 ± 0.7	1.3 ± 0.5	**<0.001**	0.297	**<0.001**	**0.001**	**<0.001**
Awakening time (h), mean ± SD	1.4 ± 0.9	1.6 ± 0.8	1.9 ± 0.9	2.0 ± 0.9	**<0.001**	**<0.001**	**0.015**	**0.006**	**0.049**
First falling asleep time (h), mean ± SD	0.9 ± 1.0	1.8 ± 2.3	2.2 ± 1.9	6.0 ± 23.1	**0.001**	**0.024**	**0.011**	0.159	0.254
Sleep efficiency (%), mean ± SD	78.9 ± 11.2	75.5 ± 10.7	73.7 ± 11.5	72.2 ± 10.8	**<0.001**	**<0.001**	0.085	**0.042**	0.092
Sleep latency (min), mean ± SD	12.6 ± 21.1	10.4 ± 19.1	10.4 ± 18.5	18.8 ± 28.7	**0.023**	0.731	**0.009**	0.185	**0.019**
Prolongation of sleep latency, No. (%)					0.201	0.718	0.100	0.380	**0.032**
No	134 (28.1)	157 (32.9)	128 (26.8)	58 (12.2)					
Yes	22 (30.1)	20 (27.4)	16 (21.9)	15 (20.5)					
Acute insomnia, No. (%)					**<0.001**	**<0.001**	**0.001**	**0.001**	**0.004**
No	60 (54.1)	34 (30.6)	15 (13.5)	2 (1.8)					
Yes	96 (21.9)	143 (32.6)	129 (29.4)	71 (16.2)					
**HRV characteristics**									
SDNN (ms), mean ± SD	117.6 ± 35.3	113.9 ± 33.4	111.3 ± 34.7	99.4 ± 29.3	**0.002**	**0.023**	**0.007**	0.051	**0.002**
SDANN (ms), mean ± SD	97.7 ± 33.1	96.3 ± 30.9	94.4 ± 31.4	82.7 ± 26.8	**0.006**	0.124	**0.005**	0.059	**0.001**
rMSSD (ms), mean ± SD	38.8 ± 23.4	30.6 ± 14.6	26.8 ± 12.4	22.7 ± 10.4	**<0.001**	**<0.001**	**<0.001**	**<0.001**	**<0.001**
pNN10 (%), mean ± SD	63.9 ± 14.4	58.4 ± 14.9	52.3 ± 15.6	45.1 ± 17.8	**<0.001**	**<0.001**	**<0.001**	**<0.001**	**<0.001**
pNN20 (%), mean ± SD	46.4 ± 18.1	39.1 ± 17.0	32.7 ± 16.5	25.2 ± 16.4	**<0.001**	**<0.001**	**<0.001**	**<0.001**	**<0.001**
pNN30 (%), mean ± SD	34.2 ± 18.8	26.5 ± 16.1	21.1 ± 14.6	17.5 ± 27.4	**<0.001**	**<0.001**	**0.001**	**<0.001**	**0.006**
pNN40 (%), mean ± SD	20.1 ± 16.9	13.5 ± 12.4	10.0 ± 10.3	6.7 ± 7.9	**<0.001**	**<0.001**	**<0.001**	**<0.001**	**<0.001**
pNN50 (%), mean ± SD	15.5 ± 15.7	9.6 ± 10.4	6.9 ± 8.5	4.5 ± 5.9	**<0.001**	**<0.001**	**<0.001**	**<0.001**	**<0.001**
ULF (ms^2^), mean ± SD	14350.2 ± 10116.2	13893.9 ± 9423.5	13558.8 ± 9542.4	10237.8 ± 7049.2	**0.016**	0.160	**0.012**	0.118	**<0.001**
VLF (ms^2^), mean ± SD	2055.4 ± 1346.2	2047.5 ± 1303.5	2011.0 ± 1212.9	1820.4 ± 1395.8	0.599	0.608	0.438	0.441	0.208
LF (ms^2^), mean ± SD	814.2 ± 607.2	768.8 ± 648.3	747.1 ± 626.9	597.0 ± 518.7	0.097	0.145	0.125	0.251	**0.044**
HF (ms^2^), mean ± SD	1003.0 ± 1330.4	629.4 ± 778.8	500.3 ± 499.4	373.8 ± 410.7	**<0.001**	**<0.001**	**0.011**	**0.007**	**0.016**
LF/HF ratio, mean ± SD	1.2 ± 0.6	1.5 ± 0.7	1.8 ± 0.8	2.1 ± 1.1	**<0.001**	**<0.001**	**<0.001**	**<0.001**	**0.004**

P1 was used to assess the comparisons between four cohorts; P2 was used to assess the comparisons between patients without and with preoperative OSA; P3 was used to assess the comparisons among mild preoperative OSA patients, moderate preoperative OSA patients, and severe preoperative OSA patients; P4 was used to assess the comparisons between mild preoperative OSA patients and moderate-to-severe preoperative OSA patients; P5 was used to assess the comparisons between mild-to-moderate preoperative OSA patients and severe preoperative OSA patients. OSA, obstructive sleep apnea; AHI, apnea-hypopnea index; SD, standard deviation; BMI, body mass index; PAS-7, perioperative anxiety scale-7; REM, rapid-eye-movement; HRV, heart rate variability; SDNN, SD of all normal RR intervals; SDANN, SD of 5 min average normal RR intervals; rMSSD, the root mean square of the successive differences; HRVTI, HRV trigonometric index; pNN10, NN10 count divided by the total number of all NN intervals; pNN20, NN20 count divided by the total number of all NN intervals; pNN30, NN30 count divided by the total number of all NN intervals; pNN40, NN40 count divided by the total number of all NN intervals; pNN50, NN50 count divided by the total number of all NN intervals; ULF, ultra-low-frequency; VLF, very-low-frequency; LF, low-frequency; HF, high-frequency. The bold values represent *p* values with statistical significance.

Adjustment by multivariate logistic regression models was conducted. Higher age [*p* < 0.001, odds ratio (OR) = 1.043], higher LF (*p* < 0.001, OR = 1.004), and higher LF/HF ratio (*p* = 0.028, OR = 1.738) were independently and positively associated with preoperative OSA; while higher pNN20 (*p* < 0.001, OR = 0.950) and higher HF (*p* < 0.001, OR = 0.998) were independently and negatively associated with preoperative OSA. Higher age (*p* = 0.005, OR = 1.024), higher VLF (*p* < 0.001, OR = 1.001), and higher LF/HF ratio (*p* = 0.003, OR = 1.655) were positively and independently associated with moderate-to-severe preoperative OSA, but higher pNN10 (*p* < 0.001, OR = 0.951) was independently and negatively associated with a lower probability of moderate-to-severe preoperative OSA. In addition, higher age (*p* = 0.003, OR = 1.036), higher VLF (*p* < 0.001, OR = 1.001), and higher LF/HF ratio (*p* = 0.013, OR = 1.516) were independently and positively associated with severe preoperative OSA, while higher pNN10 (*p* < 0.001, OR = 0.948) and higher ULF (*p* = 0.036, OR = 0.999) were independently and negatively associated with severe preoperative OSA (Table 4).

**Table 4:** Independent factors related to preoperative OSA by forward stepwise multivariate logistic regression models.

Items	*p*-value	OR	95% CI
**Model for preoperative OSA** ^**#**^			
Age (years)	<0.001	1.043	1.025–1.061
pNN20 (%)	<0.001	0.950	0.931–0.969
LF (ms^2^)	<0.001	1.004	1.002–1.005
HF (ms^2^)	<0.001	0.998	0.997–0.999
LF/HF ratio	0.028	1.738	1.060–2.849
**Model for moderate-to-severe preoperative OSA** ^*^			
Age (years)	0.005	1.024	1.007–1.041
pNN10 (%)	<0.001	0.951	0.932–0.971
VLF (ms^2^)	<0.001	1.001	1.000–1.001
LF/HF ratio	0.003	1.655	1.190–2.303
**Model for severe preoperative OSA** ^**$**^			
Age (years)	0.003	1.036	1.012–1.061
pNN10 (%)	<0.001	0.948	0.924–0.972
ULF (ms^2^)	0.036	0.999	0.999–0.999
VLF (ms^2^)	<0.001	1.001	1.000–1.001
LF/HF ratio	0.013	1.516	1.093–2.102

^#^Logistic regression model between preoperative OSA and no preoperative OSA; ^*^Logistic regression model between moderate-to-severe preoperative OSA and mild preoperative OSA; ^$^Logistic regression model between severe preoperative OSA and mild-to-moderate preoperative OSA. OSA, obstructive sleep apnea; OR, odds ratio; CI, confidence interval; pNN20, NN20 count divided by the total number of all NN intervals; LF, low-frequency; HF, high-frequency; pNN10, NN10 count divided by the total number of all NN intervals; VLF, very-low-frequency; ULF, ultra-low-frequency.

### 3.5 Correlation of clinical characteristics, sleep quality, and HRV with pNN50 and LF/HF ratio

The unadjusted correlations of preoperative OSA characteristics, clinical characteristics, sleep quality characteristics, and HRV characteristics with pNN50 and LF/HF ratio are shown in Table 5. After adjustment by multivariate linear regression models, preoperative OSA status (*b* = −3.080, *p* < 0.001) and age (*b* = −0.168, *p* < 0.001) showed independent, negative correlations with pNN50. Preoperative OSA status (*b* = 0.283, *p* < 0.001), male (*b* = 0.278, *p* < 0.001), sleep efficiency (*b* = 0.013, *p* < 0.001), and awakening time (*b* = 0.175, *p* = 0.001) showed independent, positive correlations with LF/HF ratio, while age (*b* = −0.010, *p* < 0.001) and bedtime (*b* = −0.060, *p* = 0.006) displayed independent, negative correlations with LF/HF ratio (Table 6).

**Table 5:** Correlations of pNN50 and LF/HF ratio with characteristics.

Items	pNN50		LF/HF ratio	
	*r* value	*p*-value	*r* value	*p*-value
**Preoperative OSA characteristics**				
AHI	−0.290	**<0.001**	0.398	**<0.001**
preoperative OSA status	−0.337	**<0.001**	0.388	**<0.001**
**Clinical characteristics**				
Age (years)	−0.234	**<0.001**	−0.107	**0.012**
Male	−0.063	0.140	0.281	**<0.001**
BMI (kg/m^2^)	−0.064	0.135	0.096	**0.024**
High education level	0.156	**<0.001**	0.030	0.485
Hypertension	−0.138	**0.001**	−0.020	0.645
Diabetes	−0.178	**<0.001**	0.026	0.537
PAS-7 score	0.018	0.682	−0.046	0.280
Surgical site of head or chest	0.035	0.408	0.018	0.680
**Sleep quality characteristics**				
Bedtime (h)	−0.024	0.569	−0.010	0.812
Total sleep time (h)	−0.027	0.523	0.027	0.522
Deep sleep time (h)	0.168	**<0.001**	−0.201	**<0.001**
Light sleep time (h)	−0.239	**<0.001**	0.291	**<0.001**
REM sleep time (h)	0.024	0.577	−0.075	0.078
Awakening time (h)	−0.091	**0.032**	0.076	0.074
First falling asleep time (h)	−0.044	0.320	−0.014	0.755
Sleep efficiency (%)	−0.037	0.389	0.064	0.134
Sleep latency (min)	−0.013	0.761	−0.040	0.348
Prolongation of sleep latency	0.017	0.693	−0.032	0.451
Acute insomnia	−0.115	**0.007**	0.144	**0.001**
**HRV characteristics**				
SDNN (ms)	0.480	**<0.001**	−0.109	**0.010**
SDANN (ms)	0.303	**<0.001**	−0.079	0.065
rMSSD (ms)	0.968	**<0.001**	−0.394	**<0.001**
pNN10 (%)	0.754	**<0.001**	−0.328	**<0.001**
pNN20 (%)	0.847	**<0.001**	−0.365	**<0.001**
pNN30 (%)	0.816	**<0.001**	−0.320	**<0.001**
pNN40 (%)	0.990	**<0.001**	−0.391	**<0.001**
pNN50 (%)	-	-	−0.383	**<0.001**
ULF (ms^2^)	0.347	**<0.001**	−0.101	**0.018**
VLF (ms^2^)	0.594	**<0.001**	−0.064	0.132
LF (ms^2^)	0.765	**<0.001**	−0.160	**<0.001**
HF (ms^2^)	0.804	**<0.001**	−0.353	**<0.001**
LF/HF ratio	−0.383	**<0.001**	-	-

pNN50, NN50 count divided by the total number of all NN intervals; LF, low-frequency; HF, high-frequency; BMI, body mass index; PAS-7, perioperative anxiety scale-7; REM, rapid-eye-movement; AHI, apnea-hypopnea index; OSA, obstructive sleep apnea; HRV, heart rate variability; SDNN, SD of all normal RR intervals; SDANN, SD of 5 min average normal RR intervals; rMSSD, the root mean square of the successive differences; HRVTI, HRV trigonometric index; pNN10, NN10 count divided by the total number of all NN intervals; pNN20, NN20 count divided by the total number of all NN intervals; pNN30, NN30 count divided by the total number of all NN intervals; pNN40, NN40 count divided by the total number of all NN intervals; ULF, ultra-low-frequency; VLF, very-low-frequency. The bold values represent *p* values with statistical significance.

**Table 6:** Independent factors related to pNN50 and LF/HF ratio by forward stepwise multivariate linear regression models.

Items	Unadjusted *b*	SE	Adjusted *b*	*t* value	*P*-value	VIF
**Model for pNN50 (%)**						
Preoperative OSA status	−3.080	0.503	−0.257	−6.123	<0.001	1.041
Age (years)	−0.168	0.031	−0.230	−5.493	<0.001	1.041
**Model for LF/HF ratio**						
Preoperative OSA status	0.283	0.034	0.353	8.357	<0.001	1.161
Age (years)	−0.010	0.002	−0.213	−5.284	<0.001	1.057
Male	0.278	0.062	0.183	4.473	<0.001	1.087
Sleep efficiency (%)	0.013	0.004	0.187	3.620	<0.001	1.735
Awakening time (h)	0.175	0.051	0.202	3.404	0.001	2.298
Bedtime (h)	−0.060	0.022	−0.131	−2.753	0.006	1.464

pNN50, NN50 count divided by the total number of all NN intervals; LF, low-frequency; HF, high-frequency; SE, standard error; VIF, variance inflation factor; OSA, obstructive sleep apnea.

## 4 Discussion

The current study revealed that the prevalence of preoperative OSA was 71.7%. We also identified that age, pNN20, LF, HF, and LF/HF ratio were independently associated with preoperative OSA. The linear regression analyses revealed that preoperative OSA showed an independent, negative correlation with pNN50 but an independent, positive correlation with LF/HF ratio.

OSA is considered to be a prevalent disorder affecting about 25% of all adults (23). Nevertheless, the detailed prevalence of OSA can vary greatly depending on the studied population, region, and screening method for OSA. For instance, a study conducted in the southern region of China uses a photoelectric reflector sensor evaluating the pulse oxygen saturation to screen OSA in 3,650 adults; the data shows that 30.7% of subjects have OSA (24). This study also uses a sleep questionnaire to screen OSA, and the prevalence of sleep questionnaire-screened OSA is 42.8% (24). A meta-analysis reviews eight studies with 11,009 subjects in India (mean age ranges from 35.5 years to 47.8 years) and concludes that the pooled prevalence of OSA is 11% in total adults, 13% in males, and 5% in females (25). Another meta-analysis reviewed 98 articles, which shows that the estimated prevalence of OSA was 54%; however, heterogeneity exists among the studies (26). Nevertheless, the information on the prevalence of preoperative OSA is not sufficient. On the other hand, patients with OSA about to receive surgery under general anesthesia may encounter difficulty in airway management and postoperative complications such as pulmonary complications, cardiovascular complications, delirium, and even mortality; meanwhile, the type and dose of anesthetics should be carefully considered since different anesthetics have different effect on respiratory system, and patients with OSA are at high risk of respiratory depression (27). Under this scenario, it is critical to evaluate preoperative OSA.

The current study utilized CPC to screen preoperative OSA, which was characterized by convenience and high screening accuracy (17–19). Another advantage of CPC is that it may have less first-night effect. The first-night effect refers to the reduced sleep quality when a subject moves to an unfamiliar environment; it may also occur when wearing complicated equipment for the first time when sleeping (28). It is supposed that the first-night effect could interfere with the diagnosis of OSA (29). Considering the equipment of CPC is more simplified compared with polysomnography, it may induce less first-night effect, thus promoting the screening of preoperative OSA. The data showed that by CPC screening, the prevalence of preoperative OSA was 71.7%, which was higher compared with previous studies (24–26). The probable reasons were supposed as follows: (1) The screening method for OSA in the current study might be different from previous studies. In the current study, CPC was used to screen OSA, which was characterized by high sensitivity and accuracy (30–32). (2) Patients about to receive surgery under general anesthesia might have negative emotions such as fear of surgery, concerns about surgical outcomes, etc., which could induce the activation of sympathetic nervous system and subsequently lead to preoperative OSA (13, 14). These data suggested that preoperative OSA was quite prevalent in patients about to receive surgery under general anesthesia, and CPC was feasible to screen preoperative OSA in these patients.

Identifying the factors associated with preoperative OSA could be meaningful to improve the management of preoperative OSA. The current study included the clinical characteristics, sleep quality characteristics, and HRV characteristics, then used forward stepwise multivariate logistic to analyze the independent factors associated with preoperative OSA. The data revealed that higher age, higher HF, higher LF/HF, lower pNN20, and lower HF were independently associated with a higher probability of preoperative OSA. The possible explanations are as follows: (1) Age is a well-recognized risk factor for OSA (1). The airway of individuals of higher age might collapse more easily to due to loss of collagen (33). (2) pNN20 was a time-domain parameter of the HRV, which might reflect the activity of parasympathetic nervous system (34, 35). LF, HF, and LF/HF ratio are frequency-domain parameters of HRV, which reflect the balance of sympathetic and parasympathetic nervous system (36). While the imbalance of sympathetic and parasympathetic nervous system is associated with OSA (37–39). Our findings also suggested that age and imbalance of sympathetic and parasympathetic nervous system were also independently associated with a higher probability of moderate-to-severe and severe preoperative OSA. These findings implied that additional perioperative care or monitoring should be given in subjects with higher age or imbalance of sympathetic and parasympathetic nervous system. The current study measured SDNN and SADNN due to the following reason: SDNN and SADNN are vital parameters in the HRV analysis, which could reflect the function of sympathetic and parasympathetic nervous system.

To further verify the association of preoperative OSA with the imbalance of sympathetic and parasympathetic nervous system, the current study conducted linear regression analyses. The data revealed that preoperative OSA showed an independent, negative correlation with pNN50 but an independent, positive correlation with LF/HF ratio. This finding further revealed the intercorrelation between preoperative OSA and the imbalance of sympathetic and parasympathetic nervous systems. Moreover, it was found that age showed an independent, negative correlation with pNN50; male, sleep efficiency, and awakening time presented independent, positive correlations with LF/HF ratio, while age and bedtime possessed independent, negative correlations with LF/HF ratio. The positive correlation of male with LF/HF ratio suggested the positive association of male with the activity of sympathetic nervous system, which was partly in accordance with previous studies (40, 41). These data further provide potential evidence for improving the management of preoperative OSA in patients about to receive surgery under general anesthesia.

The current study detected SDNN and SADNN due to their importance and application in the HRV analysis. Both SDNN and SDANN are important parameters for assessing the function of the cardiac autonomic nervous system. The autonomic nervous system is essential for maintaining the body’s physiological homeostasis and adapting to environmental changes, and HRV, as an indirect reflection of autonomic nervous system activity, can provide important information about the health and functional status of the heart. Specifically, SDNN and SDANN are able to reflect changes in heart rate over different time scales. SDNN mainly reflects the HRV over a shorter period of time, such as the change in heartbeat intervals per minute, while SDANN focuses on the average change in HRV over a longer period of time, such as 24 h. By measuring these two indicators, we can fully understand the overall characteristics and the changes in different time scales of HRV, so as to more accurately assess the functional status of the cardiac autonomic nervous system (42).

Several limitations in the current study should be clarified. First, the difference in the type of surgery might affect the findings of this study. Second, the current study excluded subjects with chronic sleep difficulty; thus, the findings of this study could not be applied to these individuals. Third, the current study did not evaluate the postoperative complications and their associations with preoperative OSA, which might be evaluated in further studies. Fourth, the current study did not assess the pre-existing OSA or the risk of OSA by STOPBANG criteria in the patients before the initiation of this study, which could be a bias. Fifth, the current study used CPC for diagnosing OSA due to its convenience and feasibility for assessing OSA before the night of surgery. While the gold standard for OSA diagnosis is using polysomnography, and our findings should be further validated. Sixth, the current study was a cross-sectional study and did not evaluate the surgical outcomes, including complications. The impact of preoperative OSA on the outcomes of surgery should be investigated in further studies.

Collectively, preoperative OSA is prevalent, and it is associated with age and imbalance of sympathetic and parasympathetic nervous system. The findings of this study suggest that CPC is helpful for screening preoperative OSA and its risk factors, which may promote the management of preoperative OSA.

## Data availability statement

The original contributions presented in the study are included in the article/Supplementary material, further inquiries can be directed to the corresponding authors.

## Ethics statement

This study was approved by the Ethics Committee of Hebei Provincial Hospital of Traditional Chinese Medicine (No. HBZY2020-KY-067-02). The studies were conducted in accordance with the local legislation and institutional requirements. The participants provided their written informed consent to participate in this study.

## Author contributions

SH: Conceptualization, Formal analysis, Investigation, Methodology, Validation, Writing – original draft, Writing – review & editing. GZ: Conceptualization, Formal analysis, Investigation, Methodology, Validation, Writing – original draft, Writing – review & editing. XL: Formal analysis, Methodology, Validation, Writing – review & editing. CW: Formal analysis, Methodology, Validation, Writing – review & editing. JL: Investigation, Methodology, Writing – review & editing. WH: Conceptualization, Methodology, Project administration, Supervision, Writing – review & editing. LK: Conceptualization, Methodology, Project administration, Supervision, Writing – review & editing.
