# Supplementary material for: Screening of preoperative obstructive sleep apnea by cardiopulmonary coupling and its risk factors in patients with plans to receive surgery under general anesthesia: a cross-sectional study
Source: Front Neurol. 2024 Jul 24;15:1370609. doi: 10.3389/fneur.2024.1370609 (PMC11303281; doi:10.3389/fneur.2024.1370609)
Supplement: Supplementary file 1 [file Data_Sheet_1.docx]

***Supplementary Materials***

***
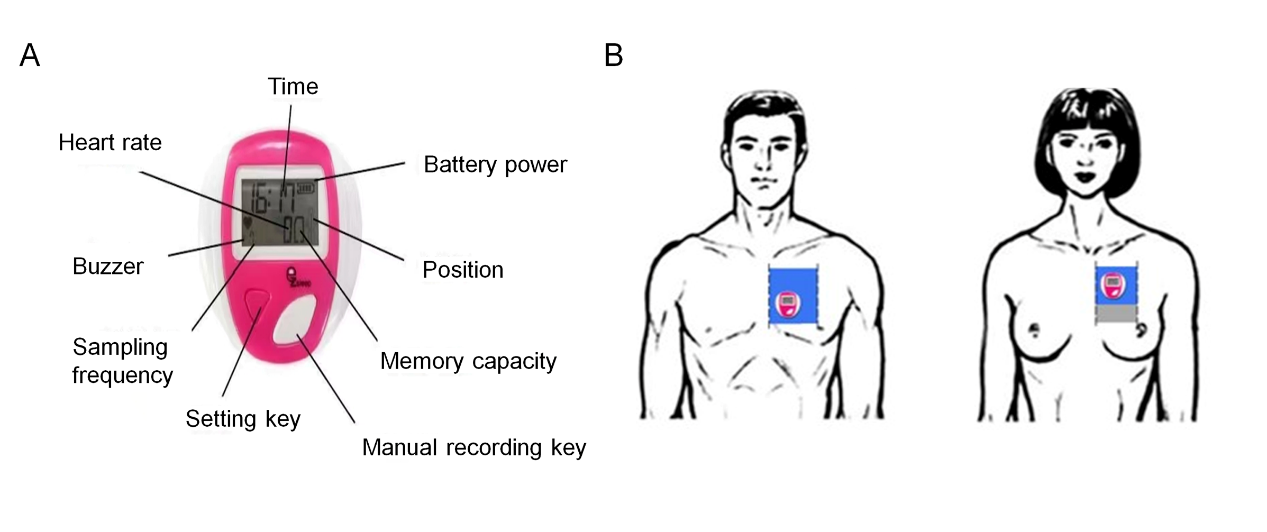
***

**Supplementary Figure 1.** Illustration of CPC. Illustration of the content displayed and buttons of CPC (**A**). Illustration of the position of wearing CPC (**B**).

**
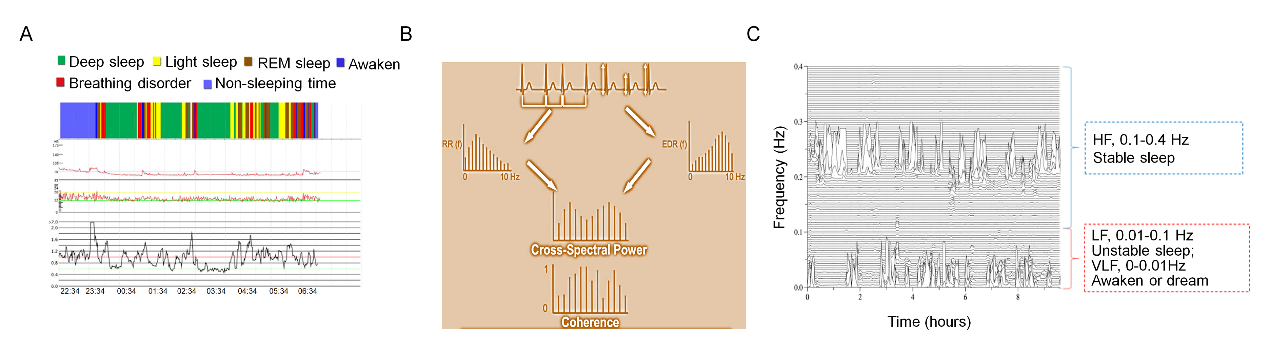
**

**Supplementary Figure 2.** Illustration of data generated by CPC. Illustration of sleep quality characteristics (**A**), time-domain parameters of HRV (**B**), and frequency-domain parameters of HRV (**C**).
